# A giant spontaneous subcapsular hematoma of the liver revealing a COVID-19 infection, a coincidence? (A case report)

**DOI:** 10.11604/pamj.2021.38.142.28172

**Published:** 2021-02-08

**Authors:** Anisse Tidjane, Amel Laredj, Nabil Boudjenan-Serradj, Salim Bensafir, Benali Tabeti

**Affiliations:** 1 Department of Medicine, Faculty of Medicine, University Oran 1, Oran, Algeria,; 2 Department of Hepatobiliary Surgery and Liver Transplantation, EHU-1st November 1954, Oran, Algeria,; 3 Department of Pediatric Infectious Disease, EHS-Canastel, Oran, Algeria,; 4 Department of Anesthesia and Surgical Reanimation, EHU-1st November 1954, Oran, Algeria

**Keywords:** SARS-CoV-2, infectious pandemic, hemorrhage, angiotensin-converting enzyme 2, case report

## Abstract

Hemorrhagic manifestations during COVID-19 infections are increasingly described in the literature. We report the first case of spontaneous subcapsular hematoma of the liver revealing a COVID-19 infection in a 44-year-old woman with no underlying health condition history, a computerized tomography evaluation showed an aspect of lung ground-glass opacities, with moderate impairment estimated at about 20%. Reverse transcription-polymerase chain reaction confirmed the diagnosis of COVID-19 infection. During the COVID-19 pandemic, non-traumatic bleeding such as spontaneous hematomas in patients with no coagulation disorder could be a manifestation of COVID-19 infection.

## Introduction

Since the declaration of the first cases of infections with severe acute respiratory syndrome coronavirus 2 (SARS-CoV-2), the risk of developing thromboembolic pathologies was obvious, hence the systematic use of preventive anticoagulation in all patients with severe disease [1]. Recently, several publications have reported cases of hemorrhagic events in many patients [2]. In this publication, we report the case of a patient with a giant sub-capsular hematoma of the liver revealing a SARS-CoV-2 infection.

## Patient and observation

**Figure 1:**
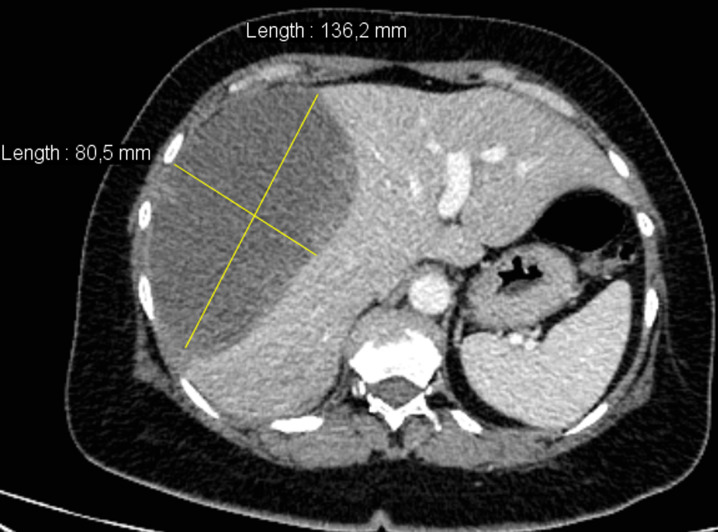
CT-scan showing sub-capsular hepatic hematoma, developed at the expense of segments VI, VII, and VIII of 136 x 80 mm in diameter

A 44-year-old Algerian woman, without any personal medical or trauma history, nor under any medical treatment, was referred to our hepatobiliary surgery department for management of a sub-capsular hematoma of the right liver developing in a context of fever and body aches dating back for more than 10 days. When questioning the patient, she describes that the onset of her illness was marked by the appearance of fever and aches followed few days later by severe pain in the right hypochondrium. The patient, however, did not report any particular respiratory signs neither notion of trauma or fall. On the day of her admission, the patient was in general condition scored WHO 2, the blood pressure was 132/80 mmHg with a heartbeat rate of 105 beats/min, and her temperature was 38.9°C. On physical examination, palpation of the right hypochondrium was very painful, the remainder of the physical examination was normal. Blood laboratory tests found: blood hemoglobin at 10.1 g/dl, hyper leukocytosis at 13,370 elm/ml with a predominance of polynuclear cells (8300 elm/ml), without lymphopenia (3400 elm/ml), a platelet count at 276,000 elm/ml, hepatic transaminases were slightly elevated with aspartate transaminase at 76 IU/ml and alanine transaminase at 72 IU/ml, normal hemostasis with a prothrombin ratio of 87% , KCT = 32.1 '' (t = 30 '') and an International Normalized Ratio of 1.10. An abdominal computerized tomography (CT-scan) performed before admission (9 days before) confirmed the presence of a giant sub-capsular hepatic hematoma, developed at the expense of segments VI, VII, and VIII of 136 x 80 mm in diameter (Figure 1).

**Figure 2:**
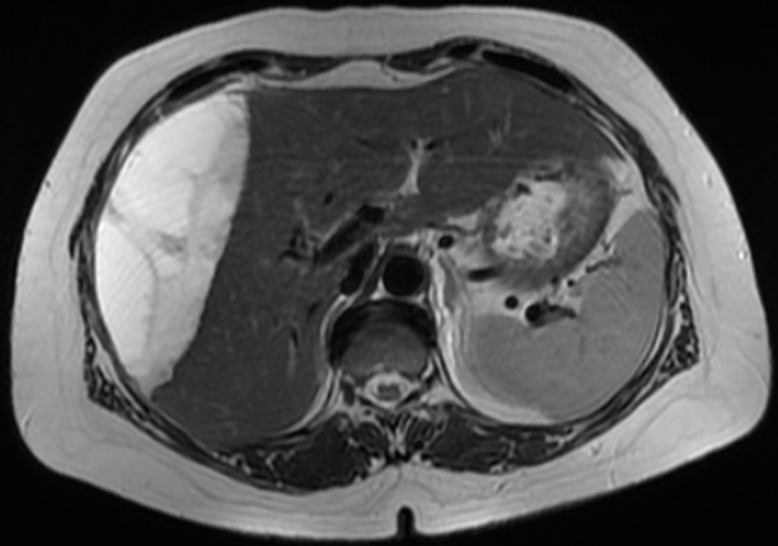
MRI showing giant sub-capsular hematoma of the liver without any traumatic or hepatic tumour pathology

**Figure 3:**
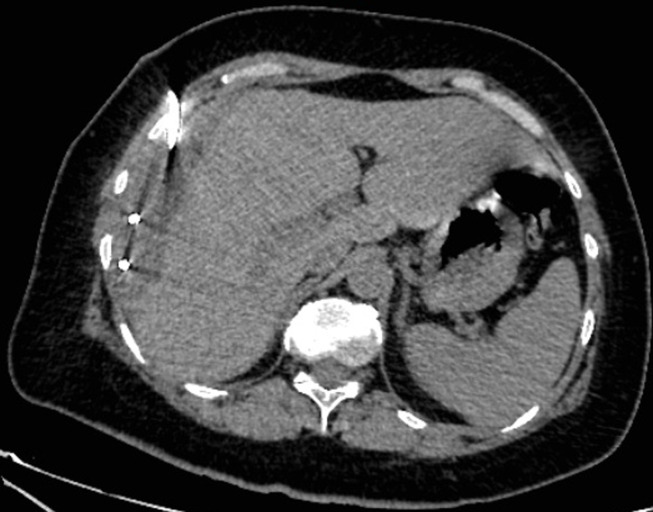
CT-scan at the first day after drainage

**Figure 4:**
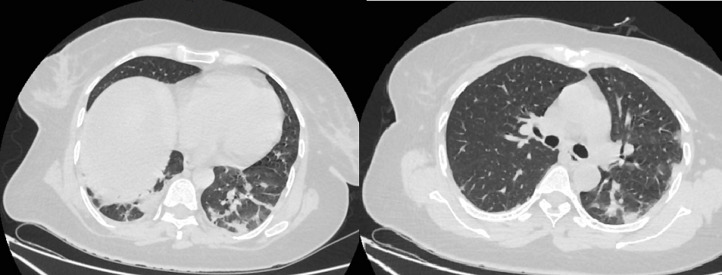
thoracic CT-scan showed lesions compatible with a SARS-CoV-2 infection (ground glass opacities)

An abdominal magnetic resonance imaging (MRI) was done urgently on the day of admission to our department and confirmed that it was a giant sub-capsular hematoma of the liver at a phase of resorption without any traumatic or hepatic tumour pathology that could explain this spontaneous bleeding (Figure 2). We performed ultrasound-guided drainage (G10 diameter nephrostomy drain) of the hematoma under local anaesthesia; the drain returned 800 ml of sequestered blood upon placement, and the bacteriological study of the collected fluid showed no bacterial contamination. On the first day after drainage, an abdominal CT-scan was performed to check the position of the drain and consider its mobilization (Figure 3); this time radiological signs in lung cuts were compatible with a SARS-CoV-2 infection. The thoracic CT-scan showed lesions compatible with a SARS-CoV-2 infection (ground glass opacities with moderate pulmonary involvement estimated between 15 and 20% as shown in (Figure 4). After review of the old radiological examinations of the abdomen, the same images were found in the pulmonary bases but not reported. A nasopharyngeal sample was sent for reverse transcription-polymerase chain reaction (RT-PCR), the result was positive for Gene N and Gene E and confirmed the presence of a SARS-CoV-2 infection. Following the Algerian national protocol, we prescribed Azithromycin 500 mg on the first day then 250 mg for the next 4 days and Hydroxychloroquine 200 mg 3/day for 10 days. The outcomes were simple with drying up on day 02; the patient became afebrile on day 03 with an improvement of its general condition after removal of the drain. She was discharged on day 07 with prescription of home confinement for 14 days.

## Discussion

On March 11^th^, 2020, WHO declares SARS-CoV-2 infection as a pandemic [3]. Patients with this disease appear to have a high risk of developing accidents of arterial and venous thrombosis [1,4]. Faced with these new data, some intensive care centers have systematically preferred to prescribe thromboprophylaxis at a therapeutic dose for their patients [4,5]. Recently, authors bring cases of hemorrhagic accidents in patients with SARS-CoV-2 infection, Fraissé *et al*. have reported an incidence of bleeding accidents in patients admitted in intensive care unit of 21% in a series of 92 cases [2]. Several publications have reported cases of hemorrhages in patients with this viral disease, cerebro-meningeal attacks are the most described, these patients´ presented neurological symptoms on admission, or even a coma, in these same patients no thromboprophylaxis was given before admission [6-8]. Other organs are also affected and cases of intramural hematoma of the aorta, bilateral adrenal hematomas, hemorrhagic cardiac tamponade, and submacular hemorrhages have been reported in the literature. In most of these cases, bleeding revealed the SARS-CoV-2 infection [9-12].

Our patient is the first case to be reported in the literature of hepatic hemorrhage in a patient with concomitant SARS-CoV-2 infection, with no notion of trauma or anticoagulation therapy. MRI was performed to look for an invisible tumor on the CT-scan, which could explain this bleeding. This examination, though expensive, was necessary because the patient had initially no evident cause that could explain the occurrence of a giant subcapsular hematoma of the liver. Liver hemorrhages caused by adenoma or hepatocellular carcinoma are reported in the literature [13]. With the presence of fever and hyperleukocytosis, first, we suspected an infection of the hematoma, hence our decision to perform urgently radiological drainage of this fluid collection, but ultimately the bacteriological study was negative.

The disease was not suspected until control CT-scan showed signs of concomitant viral infection on chest images, and SARS-CoV-2 infection was confirmed by RT-PCR. Theories tried to explain the occurrence of this hemorrhage which seems to be able to affect several organs, the most plausible is that linked to the use of the angiotensin-converting enzyme 2 receptor (ACE2-R) by the SARS-CoV-2 virus to penetrate the host cells, this binding blocks expression of ACE2-R and maybe increases intra-vascular pressure leading to hemorrhages [14]. The liver, like other organs, has ACE2-R, the inactivation of these receptors by the virus may explain the occurrence of hepatic bleeding in our patient, it should be remembered that our patient has no history of trauma and with normal hemostasis and normal platelet count, but it should be noted that this remains a hypothesis that requires investigation to be confirmed [15]. It is currently impossible to establish the cause-and-effect relationship between the SARS-CoV-2 infection and this hepatic hematoma, but it seems important to us to suspect this link and to publish it to contribute to a better understanding of the effect of this new viral infection.

## Conclusion

Hemorrhagic manifestations may be indicative of a viral SARS-CoV-2 infection, non-traumatic bleeding such as spontaneous hematomas could be a manifestation of SARS-CoV-2 infection. Our described case is the first case of spontaneous subcapsular hematoma of the liver revealing a SARS-CoV-2 infection.
